# Palladium Nanoparticle-Loaded Mesostructural Natural Woods for Efficient Water Treatment

**DOI:** 10.3390/polym15030658

**Published:** 2023-01-27

**Authors:** Zirun Wang, Chao Jia, Hengxue Xiang, Meifang Zhu

**Affiliations:** State Key Laboratory for Modification of Chemical Fibers and Polymer Materials, College of Materials Science and Engineering, Donghua University, Shanghai 201620, China

**Keywords:** wood, Pd nanoparticles, water treatment, catalytic degradation

## Abstract

Natural wood with oriented microchannels and unique multi-level structures is an ideal candidate for making water treatment membranes. Here, palladium nanoparticles are loaded into different kinds of natural woods and the degradation property of the wood membranes for organic pollutants are investigated. The water flux of hardwoods is significantly higher than that of softwood due to the existence of large vessel elements. For the single pollutant, both hardwood and softwood show high degradation efficiency for methylene blue and methylene orange, while the degradation efficiency of the softwoods for 4-nitrophenol is significantly higher than that of the hardwoods due to their lower water flux. For the mixed pollutants, all the wood membranes have a good degradation property for different concentrations of methylene blue in polluted water, while the degradation efficiency of high concentration methylene orange and 4-nitrophenol is low. Our work will provide some guidance for the degradation of organic pollutants in actual polluted water.

## 1. Introduction

With the development of industry, more and more wastewater has been produced and discharged into the environment. The wastewater, especially the wastewater containing organic pollutants, will cause serious pollution to the environment and harm to the human body [1,2]. The removal of organic pollutants from wastewater is essential to the protection of the environment and human health. Several methods have been developed to remove organic pollutants, including physical adsorption [3,4,5,6], chemical oxidation [7,8], membrane filtration [9,10,11,12], etc. However, these treatment methods still have some problems, including low efficiency, poor durability, secondary pollution and so on. Therefore, it is urgent to develop water treatment materials with high efficiency, durability and no secondary pollution.

Wood, as a kind of natural renewable material, has attracted more and more attention in recent years [13,14,15,16] and has been applied in many fields, including transparent papers [17,18], solar steam generation [19,20], etc. Natural wood has oriented microchannels, unique multi-level structures and a large number of active groups, which can be physically or chemically modified to obtain various functional materials. For example, the porous structure of natural wood can be used to obtain highly efficient oil adsorption materials through delignification treatment [21]. Studies have also shown that efficient water purification can be achieved by loading metal nanoparticles into the microchannels within natural wood [22,23]. However, the treatment effect of natural wood loaded with metal nanoparticles on the mixture of organic pollutants needs to be further studied.

Natural wood can be divided into broadleaf wood and coniferous wood, which are called hardwood and softwood, respectively [24,25]. Hardwoods are composed of fiber tracheids and vessel elements, which account for approximately 20% and 50% of wood pore volume, respectively. The vessel elements have a larger diameter than the fiber tracheids. Generally, the vessel elements are connected by perforated plates with micron holes at the cell ends, which allow water to continuously travel along the wood microchannels. Unlike hardwoods, softwoods have only fiber tracheids for transporting water and ions. The significantly different structures of hardwoods and softwoods result in different physical properties [26,27] and functional materials made from them. Therefore, it is of great significance to study the effect of pore structures of natural woods on their water treatment performance.

In this work, we loaded palladium nanoparticles into different kinds of natural woods and prepared wood membranes (palladium nanoparticle-loaded natural wood membranes, PNNW membranes) with high efficiency for water treatment. Three hardwoods, including basswood, beech wood and balsa wood, and two softwoods, including pine wood and fir wood, were selected as the base materials. The effect of the microstructures of the different natural woods on the catalytic degradation of organic pollutants by the wood membranes was investigated. In addition, we also studied the degradation performance of wood membranes on mixtures of different organic pollutants to evaluate their practical application effect. This work will provide some guidance for the application of natural wood in practical water treatment.

## 2. Experiment

### 2.1. Materials and Chemicals

Palladium chloride (PdCl_2_), methylene bromide (MB), methylene orange (MO), 4-nitrophenol (4-NP), sodium borohydride (NaBH_4_), hydrochloric acid (HCl) and sodium hydroxide (NaOH) were purchased from National Pharmaceutical Group Corporation (Shanghai, China). Basswood (*Tilia*), beech wood (*Fagus sylvatica* L.), balsa wood (*O. pyramidale*), pine wood (*Pelargonium hortorum*) and fir wood (*Pseudotsuga sinensis* Dode) were purchased from Anhui Meiru Company (Hefei, China). These wood samples were taken from healthy parts of mature wood.

### 2.2. Preparation of PNNW Membranes

PdCl_2_ solution (0.5–2 mg mL^−1^) was prepared by adding PdCl_2_ powders (50–200 mg) and HCl (1 mL, 2 mol L^−1^) to deionized water (100 mL), and heating at 60 °C for 1 h. Natural wood membranes with a dimension of 8 mm × 50 mm × 50 mm (thickness × length × width) were immersed in PdCl_2_ solution and heated at 40–100 °C for 4–14 h to obtain PNNW membranes. Unless otherwise stated, the PNNW membranes for dye degradation property test were prepared by heating natural wood in 2 mg mL^−1^ PdCl_2_ solution at 80 °C for 12 h.

### 2.3. Water Flux Test

The water flux of the PNNW membranes was calculated by testing the time it took 1 L of water to pass through a certain area of wood membranes at a pressure of 0.08 MPa. The water flux (F, L m^−2^ h^−1^) was obtained using the following equation:

F = V/(A × T)

where V represents the volume of filtered water (1 L), A (m²) represents the effective area of PNNW membranes and T (h) is the time for water to pass through the membranes.

### 2.4. Dye Degradation Property Test

The degradation of methylene blue, methyl orange and 4-nitrophenol in the presence of NaBH_4_ was used to evaluate the water treatment performance of PNNW membranes. 50 mg NaBH_4_ and different amounts of dyes were mixed with deionized water to obtain 500 mL aqueous solution. For mixed solutions containing two or three dyes, the concentration of each dye is the same. The solutions then flowed through the PNNW membrane to degrade the organic dyes. The pH value of the solutions was adjusted using HCl and NaOH. The absorbance measurement was performed for the solutions before and after filtration. The degradation efficiency (*η*, %) was calculated using the following equation:

*η* (%) = (C_0_ − C)/C_0_ × 100%

where C_0_ is the initial concentration of the solution and C is the concentration of the filtered solution.

## 3. Characterization

The microstructures of wood were characterized using a scanning electron microscope (JSM-5600LV, JEOL, Tokyo, Japan). The absorbance of dye solution before and after filtration was measured using a UV-vis spectrophotometer in the wavelength of 200–800 nm (UV1750 lambda 35, Mettler Toledo, Columbus, OH, USA). Wood density was calculated based on the measured mass and volume.

## 4. Results and Discussion

### 4.1. Preparation of Pd Nanoparticle-Loaded Wood Membranes and Degradation Mechanism

Natural wood is mainly composed of cellulose, hemicellulose and lignin, which account for more than 90% of the total weight of wood. Cellulose is formed by dehydration and condensation of glucoses, and mainly acts as a skeleton in wood cells. Hemicellulose is polymerized by glucoses and other kinds of monosaccharides, and its molecular weight is generally lower than that of cellulose. Hemicellulose contains a large number of branched chains, and mainly plays a filling role in wood cells. Lignin is a three-dimensional network polymer composed of p-hydroxyphenyl (H), guaiacyl (G) and syringyl (S) units. Lignin serves primarily as a binder in wood, binding cellulose and hemicellulose together.

Natural wood has excellent physical and mechanical properties. Due to the oriented microchannels, natural wood exhibits anisotropic physical and mechanical properties, including anisotropic mass transport characteristics [14] and anisotropic mechanical properties [16]. An interconnected pore network can be formed in natural wood by oriented microchannels and pits on cell walls, which is particularly suitable for liquid transport. Therefore, natural wood has been widely used for liquid-based transport applications, such as solar steam generation [16,28] and cleanup of viscous crude oil spill [29]. In addition, by loading catalysts into natural wood and treating wastewater containing organic dyes in the natural wood’s inherent microchannels, the efficient catalytic degradation of organic pollutants can be achieved, and the consumption of non-renewable resources and energy can be effectively reduced.

In order to load Pd nanoparticles into wood, wood was immersed in a palladium nanoparticle precursor solution. The lignin distributed uniformly in wood microchannels can effectively reduce Pd ions to Pd nanoparticles. Cellulose and hemicellulose contain large amounts of hydroxyl groups, which can firmly immobilize Pd nanoparticles to wood microchannels. The oriented microchannels in natural wood are conducive to water transport, and water can effectively contact Pd nanoparticles on the microchannels during transport, so as to achieve efficient degradation of organic pollutants in wastewater.

**Figure 1:**
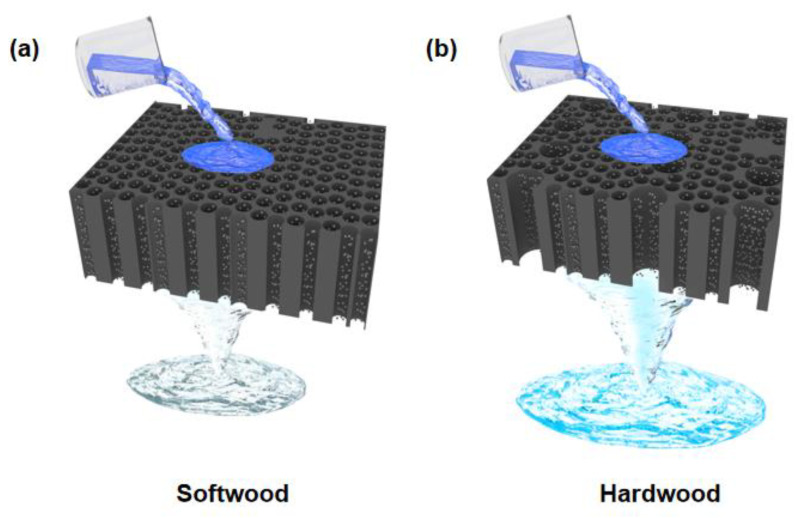
Schematic showing the catalytic degradation of pollutants in wastewater by (**a**) softwood and (**b**) hardwood loaded with Pd nanoparticles.

Natural wood has a light yellow color due to the presence of lignin components (Figure S1). When Pd nanoparticles were loaded into wood microchannels, the wood turned black (Figure S2). Softwoods have smaller diameter microchannels, which result in lower water flux. Because of the slower flow rate of water and the longer contact time between organic pollutants in water and catalysts, softwoods are generally more efficient in degradation (Figure 1a). Hardwoods have larger vessels with larger pores than softwoods, so water flux is relatively higher. However, the degradation efficiency is low due to the short contact time between organic pollutants and catalyst (Figure 1b).

### 4.2. Microstructures of Natural Woods

**Figure 2:**
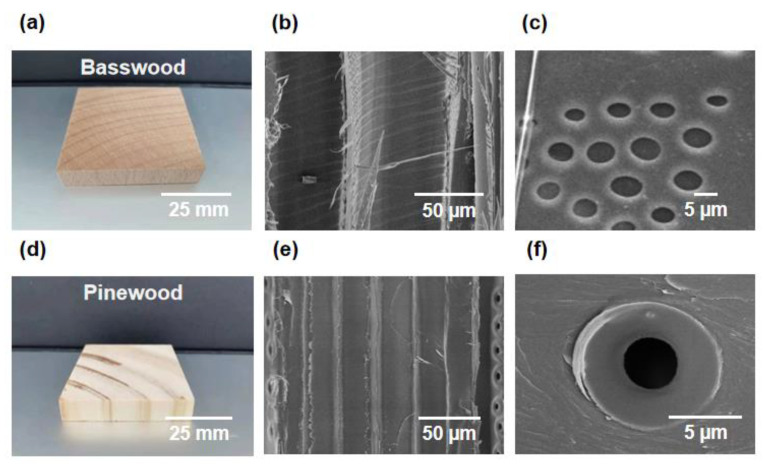
Microstructures of hardwood and softwood. (**a**) Digital image of a typical hardwood, basswood. (**b**) SEM image showing the vessels and tracheids of basswood. (**c**) SEM image of the pits on the cell walls of basswood. (**d**) Digital image of a typical softwood, pine wood. (**e**) SEM image showing the tracheids of pine wood. (**f**) SEM image of the pits on the cell walls of pine wood.

Trees rely on internally oriented microchannels to transport water and nutrients from the bottom up to support their growth. Both hardwoods and softwoods from trees have anisotropic aligned microchannels. Figure 2a,d show a typical hardwood, basswood, and a typical softwood, pine wood, respectively. It can be seen from the SEM image that basswood includes vessels with a diameter of about 100 microns and tracheids with a diameter of dozens of microns (Figure 2b). If we zoom in on the cell walls of basswood, we can see that they have a large number of pits with a diameter of several microns on their surface (Figure 2c).

In contrast to basswood, pine wood contains only tracheids, which are about 20 microns in diameter and are very uniform (Figure 2e). There are also pits on the cell walls of pine wood, but the number is significantly less than that of basswood (Figure 2f). The orientated microchannels and pits on the cell walls in natural wood form an interconnected pore network. Water can be transported not only along the microchannels but also horizontally through the pits.

**Figure 3:**
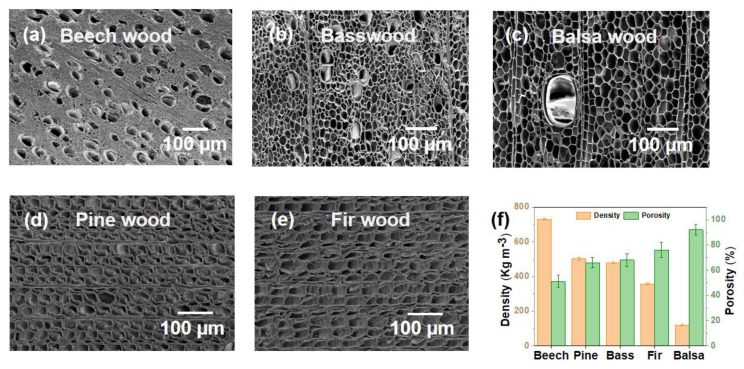
Comparison of pore structure and porosity of hardwood and softwood. SEM images of different hardwoods, (**a**) beech wood, (**b**) basswood and (**c**) balsa wood. SEM images of different softwoods, (**d**) pine wood and (**e**) fir wood. (**f**) Density and porosity of different wood species.

Pore structure and porosity have an important influence on the water treatment performance of natural wood. Figure 3a–c shows the SEM images of the upper surfaces of three different hardwoods, beech wood, balsa wood and basswood, and it can be observed that they have significantly different lumen diameters. In addition, the difference in vessel and tracheid diameter can be clearly seen from the top view of natural wood. In contrast to the complex structure of hardwoods, softwoods have only tracheids with uniform lumen diameter (Figure 3d,e), and the pore diameter of different softwoods does not differ much.

Different natural woods have different pore structures, resulting in their different densities and porosities (Figure 3f). In the case of hardwoods, the difference between pore structures of different hardwoods is very large, resulting in markedly different densities and porosity. Beech wood has a density of 731 kg m^−3^ and 51% porosity, while balsa wood, also a hardwood, has a density of 121 kg m^−3^ and a porosity of 92%. For softwoods, their density and porosity may also differ quite a lot, for example, the densities of pine wood and fir wood are 503 kg m^−3^ and 358 kg m^−3^, respectively, and their porosities are 67% and 76%, respectively. However, the difference in density and porosity between softwoods is not as great as that of hardwoods [16].

### 4.3. Degradation Property of PNNW Membranes for Single Organic Pollutant

**Figure 4:**
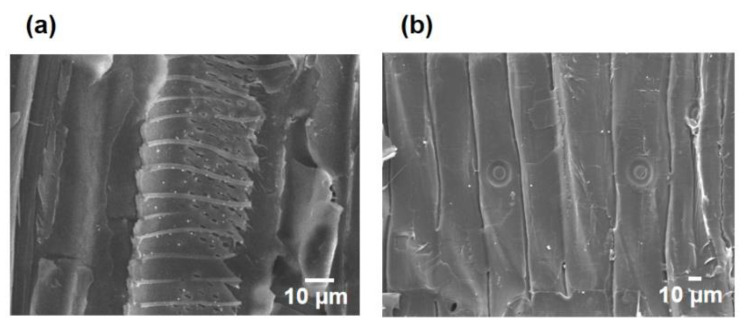
SEM images of the microchannels in the (**a**) basswood and (**b**) pine wood to show the Pd nanoparticles.

**Figure 5:**
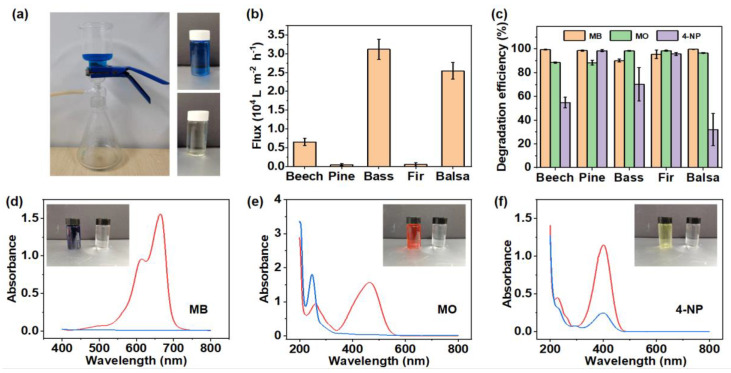
Degradation property of PNNW membranes for single organic pollutant. (**a**) Digital images showing the home-made filter device and solution before and after filtration. (**b**) Water fluxes of different natural woods. (**c**) Degradation efficiency of PNNW membranes to methylene blue (MB), methylene orange (MO) and 4-nitrophenol (4-NP) solutions with concentration of 20 mg L^−1^. Absorbance curves of (**d**) MB, (**e**) MO and (**f**) 4-NP solutions before (red curve) and after (blue curve) filtration.

The PNNW membranes were prepared by loading Pd nanoparticles into the wood microchannels. The Pd nanoparticles with a size of 30–100 nm can be clearly visualized in the wood microchannels (Figure 4). A home-made filter device was used to test the degradation property of PNNW membranes for different organic pollutants (Figure 5a). We first determined the water flux of different woods to evaluate their water treatment efficiency. The water fluxes of beech wood, basswood and balsa wood are 6541, 31,177 and 25,459 L m^−2^ h^−1^, respectively, which are much higher than those of pine wood and fir wood (Figure 5b). Note that the water fluxes of pine wood and fir wood are only 487 and 561 L m^−2^ h^−1^, respectively. The significantly higher water fluxes of hardwoods can be attributed to their large vessel elements, which enable faster water transport.

In addition, different hardwoods also exhibit significantly different water fluxes, which may be caused by the different diameters of the microchannels in different woods. Although beech wood contains a large number of vessel elements, the average diameter of its fiber tracheids is about 15 μm, significantly smaller than that of basswood and balsa wood (Figure 3a–c and Figure S3), resulting in a low water flux. Although the average diameter of fiber tracheids of balsa wood is larger than that of basswood, the number of vessel elements with a larger diameter is significantly less than that of basswood (Figure 3b,c and Figure S3), so the water flux of balsa wood is slightly lower than that of basswood.

In the textile industry, azo dyes are widely used, so we chose azo dyes, methylene blue (MB), methylene orange (MO) and 4-nitrophenol (4-NP), as model dyes to evaluate the degradation property of PNNW membranes. The absorbance of MB, MO and 4-NP solutions before and after filtration was measured to calculate the degradation efficiency of these solutions by PNNW membranes (Figure 5c–f). The characteristic absorption peaks of MB, MO and 4-NP are located at 640, 460, and 400 nm, respectively. After filtration, the characteristic absorption intensity of various organic dye solutions decreased significantly. The color of MB, MO and 4-NP solution changed from blue, orange and light yellow, respectively, to colorless, which directly reflected the degradation effect of the PNNW membranes.

Different PNNW membranes have different degradation properties for different organic dyes. All the PNNW membranes showed excellent degradation performance for MB and MO without significant difference. In particular, the degradation efficiency of beech wood, pine wood and balsa wood for MB and the degradation efficiency of basswood and fir wood for MO reached more than 99% in single dye solution. These results indicate that Pd nanoparticles have very high degradation efficiency for MB and MO. Even under the condition of the high water flux of hardwood, they can achieve a similar degradation effect to softwood with low water flux.

The degradation efficiency of 4-NP by the five PNNW membranes was quite different. The 4-NP degradation efficiencies of beech wood, pine wood, basswood, fir wood and balsa wood were 55%, 99%, 67%, 96% and 32%, respectively. The degradation efficiency of the two softwood species, pine wood and fir wood, for 4-NP was significantly higher than that of the hardwoods. These results indicate that the degradation of 4-NP by Pd nanoparticles takes a longer time than MB and MO. Since there are many large-diameter vessel elements in hardwoods, the 4-NP solution can pass through the filter membranes quickly, and the 4-NP cannot be effectively degraded by the Pd nanoparticles. However, softwoods only contain small diameter tracheids, so the water flux is low and the solution passes through the filtration membranes for a longer time. Under such a condition, 4-NP has enough time to contact Pd nanoparticles and be degraded, so the degradation efficiency is higher.

The effects of pH value, PdCl_2_ solution concentration, heat treatment temperature and time on the degradation efficiency of the basswood-based PNNW membrane for the MB solutions were investigated. The results showed that the property of PNNW membrane under alkaline conditions is better than that under acidic conditions. NaBH_4_ is weakly alkaline and easy to decompose under acidic conditions, so the degradation efficiency is low under acidic conditions (Figure S4). With the increase in PdCl_2_ solution concentration, the color of the wood membranes obtained in the same treatment conditions gradually deepened (Figure S5). The degradation efficiency remained high even with low-concentration PdCl_2_ treatment (Figure S6).

We prepared different basswood-based PNNW membranes by changing the heat treatment time (4–14 h) and temperature (40–100 °C), and investigated the effect of heating time and temperature on the degradation efficiency (Figures S7–S9). It can be seen from Figure S8 that the degradation efficiency of PNNW membranes for 20 mg L^−1^ MB solution gradually increases with the increasing heating time. When the heating time increases from 4 h to 8 h, the degradation efficiency increases rapidly from 37% to 88%. When the heating time is increased to more than 10 h, the degradation efficiency is essentially unchanged with the increase in heating time, which is about 95%. With the increase in heating time, more Pd^2+^ can be reduced to Pd nanoparticles, thus improving the catalytic property. The effect of heat treatment temperature on the catalytic degradation efficiency of PNNW membranes was small. Compared with that of PNNW membranes treated at 40 °C, the degradation efficiency of PNNW membranes treated at 100 °C was only slightly improved.

In order to verify that organic dyes are degraded by Pd nanoparticles and not by some components in the wood, we performed filtration experiments using natural wood. When the organic dye solution was filtered through natural wood, no obvious color change was observed. The characteristic absorption peaks of various dyes before and after filtration did not change clearly, as observed through infrared absorption spectrum analysis (Figure S10). These results indicate that natural wood has no degradation effect on organic dyes.

### 4.4. Degradation Property of PNNW Membranes for Mixed Organic Pollutant

**Figure 6:**
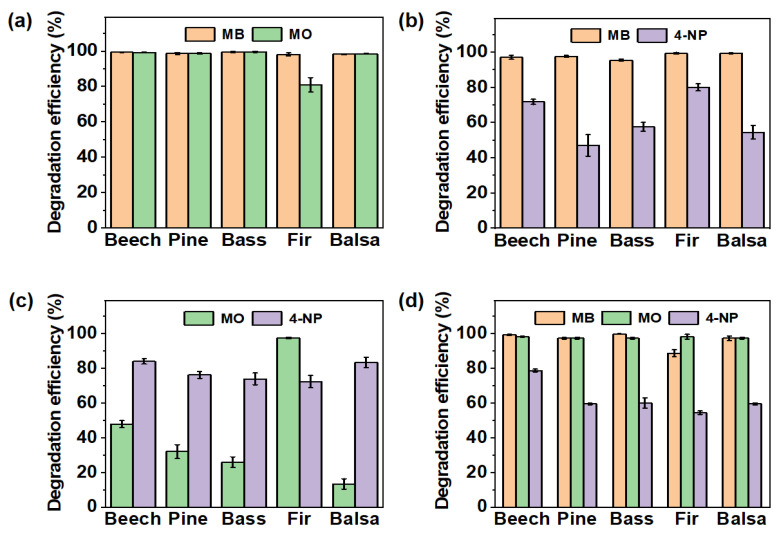
Degradation property of PNNW membranes for mixed organic pollutant. Degradation property of PNNW membranes for (**a**) MB and MO mixed solution, (**b**) MB and 4-NP mixed solution, (**c**) MO and 4-NP mixed solution and (**d**) MB, MO and 4-NP mixed solution. The concentrations of MB, MO and 4-NP in the mixed solution are 20 mg L^−1^.

Industrial wastewater usually contains a variety of organic pollutants, so it is necessary to study the degradation property of the prepared PNNW membranes for mixed organic pollutants. Therefore, we studied the degradation property of PNNW membranes for pollutants in mixed solutions containing two dyes and three dyes. For the mixed solution containing MB and MO, the PNNW membranes had high degradation efficiency for both organic pollutants in the mixed solution. The degradation efficiency of MO by fir wood-based PNNW membrane was about 80%, and the degradation efficiency of both dyes by other PNNW membranes was more than 99% (Figure 6a).

For the mixed solution containing MB and 4-NP, the degradation efficiency of the five PNNW membranes for MB in the mixed solution was more than 95%, but their degradation property for 4-NP was poor (Figure 6b). We found that all PNNW membranes had higher degradation efficiency for 4-NP in the mixed solution of MO and 4-NP. The degradation efficiency of the fir wood-based PNNW membrane for MO in the MO and 4-NP mixed solution was about 98%, and the high degradation efficiency may be caused by the longer catalytic degradation time. However, the degradation efficiency of other PNNW membranes for MO in the MO and 4-NP mixed solution was lower than 50%, and tends to decrease with the increase in wood porosity (Figure 6c).

The degradation property of PNNW membranes for the MB, MO and 4-NP mixture was further investigated. All PNNW membranes showed a degradation efficiency of more than 90% for MB and MO in the mixed solution. In particular, the beech wood-, pine wood-, basswood- and balsa wood-based PNNW membranes had a degradation efficiency of more than 98% for both pollutants. However, the degradation efficiency of all PNNW membranes for 4-NP in the mixed solution was less than 80%, similar to the situation of the two pollutants (Figure 6d). Through the above analysis, it can be concluded that the degradation property of PNNW membranes for different organic pollutants in the mixed solutions is affected by the types of pollutants.

When the dye concentration in the mixed solutions was increased from 20 mg L^−1^ to 30 mg L^−1^ and 40 mg L^−1^, the degradation property of all the PNNW membranes for MB in the mixed solutions basically did not change significantly. However, the degradation efficiencies of the MO component in the MB and MO mixture solution, 4-NP component in the MB and 4-NP mixture solution, and MO component in the MB, MO and 4-NP mixture solution were significantly reduced (Figures S11 and S12). The above results show that all PNNW membranes have a good degradation property for different concentrations of MB in polluted water, but when the concentration of MO and 4-NP in polluted water is too high, the catalysts in the wood membranes are too late to degrade these dyes, resulting in low degradation efficiency.

## 5. Conclusions

Five kinds of natural wood membranes loaded with Pd nanoparticles were prepared and the catalytic degradation efficiency of these wood membranes for single and mixed dyes was studied. Softwoods have a low water flux because they contain only small-diameter fiber tracheids. Compared with hardwood, softwood has a significantly higher degradation efficiency for single 4-NP due to sufficient contact time between the dye and catalyst. For the single MB and MO, both hardwood and softwood show high degradation efficiency. For the mixed pollutants, there are some interferences between different pollutants, resulting in different catalytic degradation efficiency. All the wood membranes have a good degradation property for different concentrations of MB, while the degradation efficiency of high-concentration MO and 4-NP is low. Therefore, the appropriate natural wood-based water treatment membrane should be selected according to the kinds of pollutants contained in the polluted water in practical applications.

## Data Availability

The data are available from the corresponding authors upon reasonable request.

## Supplementary Materials

The following supporting information can be downloaded at: https://www.mdpi.com/article/10.3390/polym15030658/s1, Figure S1: Digital image of a natural wood with light yellow. Figure S2: Digital image of a Pd nanoparticle loaded natural wood. Figure S3: Distribution of pore diameter of different woods. Figure S4: Catalytic degradation prop-erty of basswood based PNNW membrane to MB solution under different pH conditions. Figure S5: PNNW membranes prepared using PdCl2 solutions with different concentrations. Figure S6: Catalytic degradation efficiency of the basswood based PNNW membranes treated with different PdCl2 concentra-tions to 20 mg L^−1^ MB solution. Figure S7: PNNW membranes prepared with different heating time. Figure S8: Catalytic degradation efficiency of the basswood based PNNW membranes treated with different heat-ing time for 20 mg L^−1^ MB solution. Figure S9: Catalytic degradation efficiency of basswood based PNNW membrane treated with different heating temperatures for 20 mg L^−1^ MB solution. Figure S10: Absorbance curves of MB, MO and 4-NP solutions before and after filtration using natural wood. Figure S11: Degrada-tion property of PNNW membranes to mixed organic pollutant (The concentrations of MB, MO and 4-NP in the mixed solution are 30 mg L^−1^). Figure S12: Degradation property of PNNW membranes to mixed organic pollutant (The concentrations of MB, MO and 4-NP in the mixed solution are 40 mg L^−1^).
