# Supplementary material for: Palladium Nanoparticle-Loaded Mesostructural Natural Woods for Efficient Water Treatment
Source: Polymers (Basel). 2023 Jan 27;15(3):658. doi: 10.3390/polym15030658 (PMC9919286; doi:10.3390/polym15030658)
Supplement: Supplementary file 1 [file polymers-15-00658-s001.zip › polymers-2056105-supplementary.pdf]

Supplementary Information

Palladium Nanoparticle Loaded Mesostructural Natural Woods for Efficient Water Treatment

Zirun Wang [1], Chao Jia [1,*], Hengxue Xiang [1,*], Meifang Zhu

[1] State Key Laboratory for Modification of Chemical Fibers and Polymer Materials, College of Materials Science and Engineering, Donghua University, Shanghai 201620, China

[*] **Email:** hengxuexiang@dhu.edu.cn; jiachao0806@dhu.edu.cn

Supplementary Figures

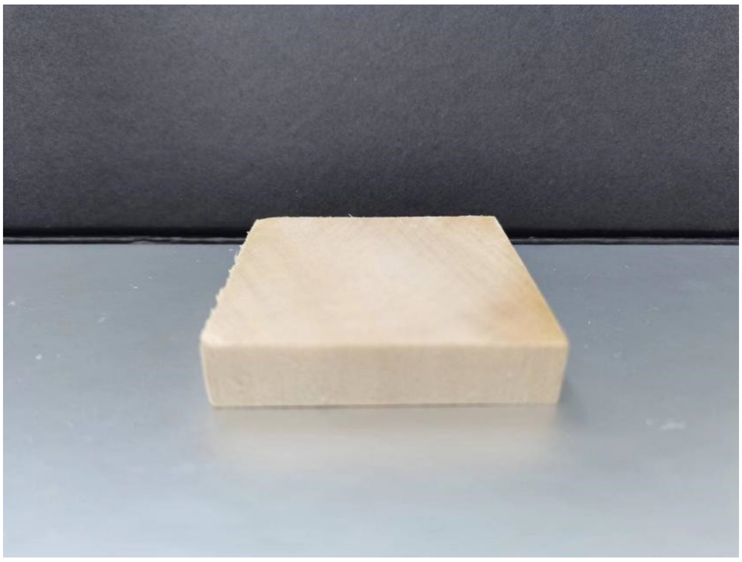

Figure S1. Digital image of a natural wood with light yellow.

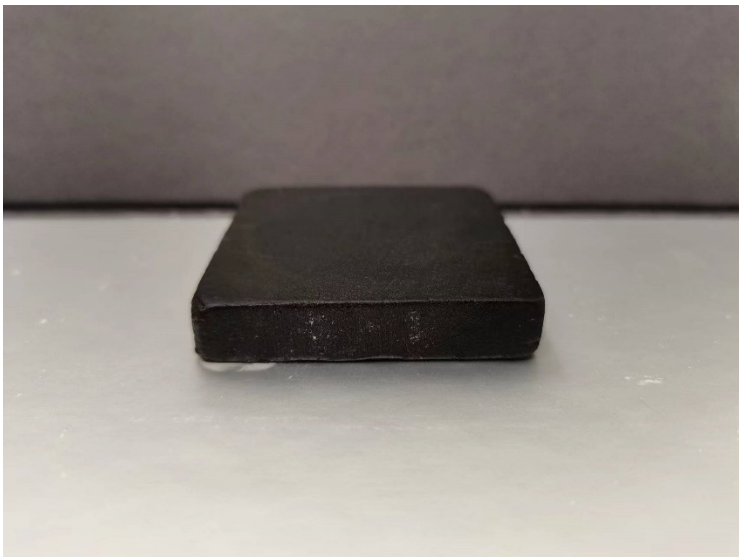

Figure S2. Digital image of a Pd nanoparticle loaded natural wood, which is black due to the presence of Pd nanoparticles.

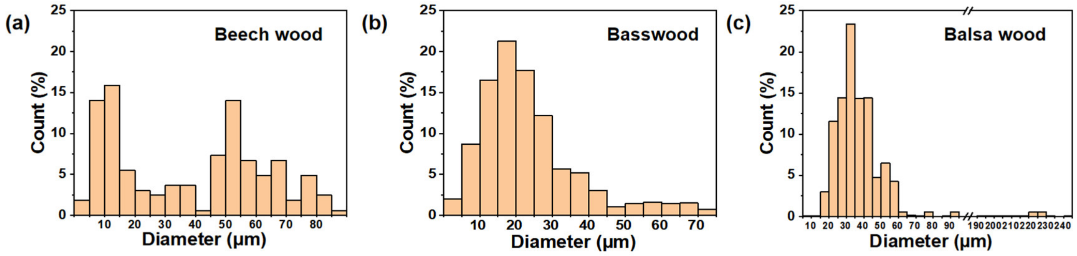

Figure S3. Distribution of pore diameter of different woods.

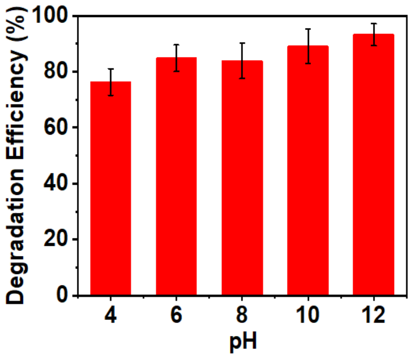

Figure S4. Catalytic degradation property of basswood based PNNW membrane to MB solution under different pH conditions

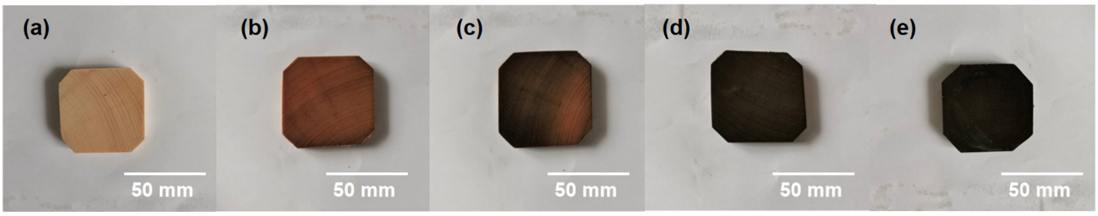

Figure S5. PNNW membranes prepared using $PdCl_2$ solutions with different concentrations. Digital images of (a) natural basswood, and PNNW membranes obtained by treatment of $PdCl_2$ solutions with concentration of (b) 0.5 mg mL^{-1}, (c) 1 mg mL^{-1}, (d) 1.5 mg mL^{-1}, (e) 2 mg mL^{-1}.

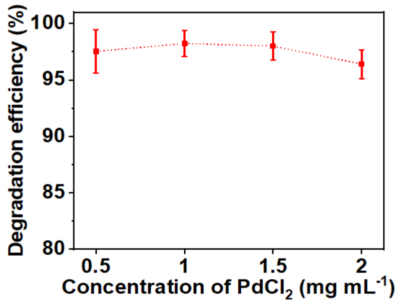

Figure S6. Catalytic degradation efficiency of the basswood based PNNW membranes treated with different $PdCl_2$ concentrations to 20 mg L^{-1} MB solution.

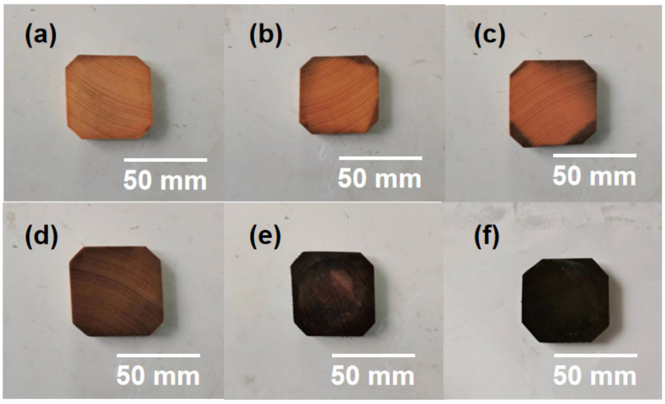

Figure S7. PNNW membranes prepared with different heating time. Digital images of PNNW membranes obtained by heat treatment time of (a) 4 h, (b) 6 h, (c) 8 h, (d) 10 h, (e) 12 h, (f) 14 h.

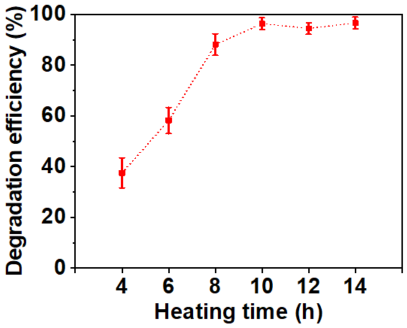

Figure S8. Catalytic degradation efficiency of the basswood based PNNW membranes treated with different heating time to 20 mg L^{-1} MB solution.

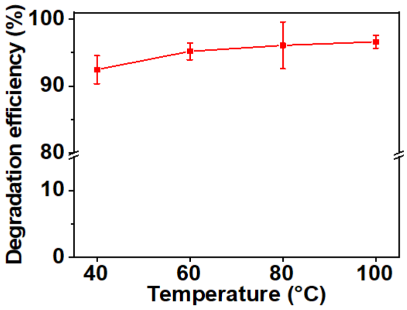

Figure S9. Catalytic degradation efficiency of basswood based PNNW membrane treated with different heating temperatures for 20 mg L^{-1} MB solution.

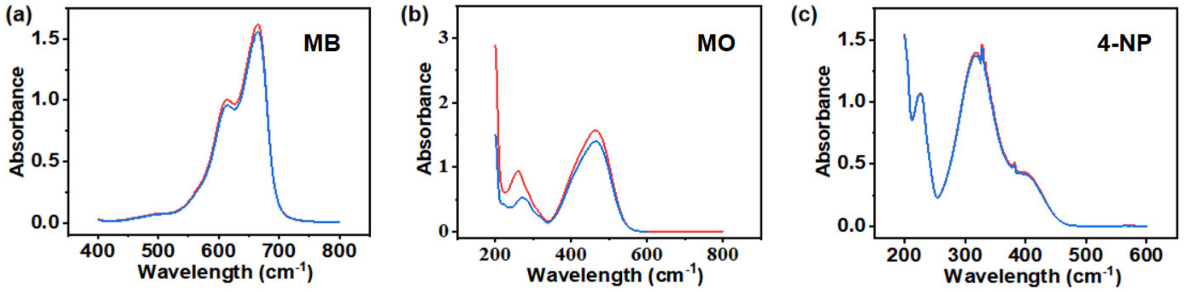

Figure S10. Absorbance curves of (a) MB, (b) MO and (c) 4-NP solutions before and after filtration using natural wood.

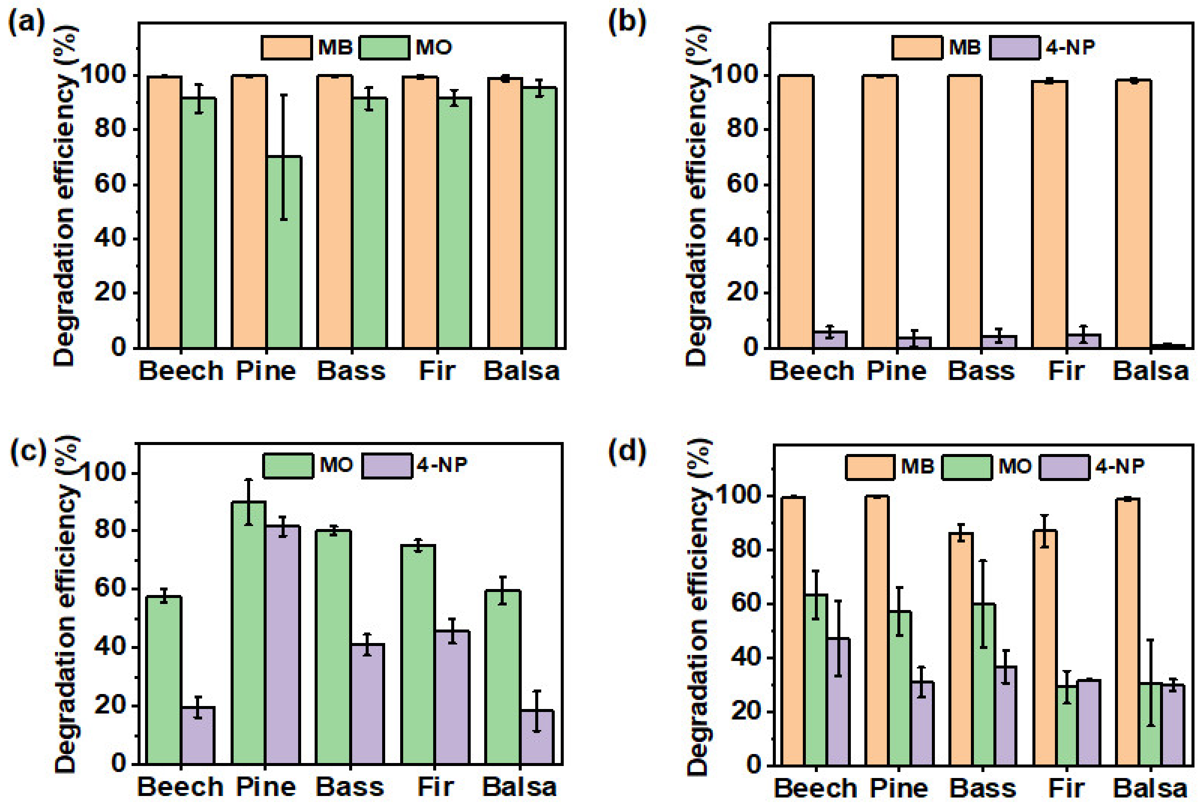

Figure S11. Degradation property of PNNW membranes to mixed organic pollutant. Degradation property of PNNW membranes to (a) MB and MO mixed solution, (b) MB and 4-NP mixed solution, (c) MO and 4-NP mixed solution, (d) MB, MO and 4-NP mixed solution. The concentrations of MB, MO and 4-NP in the mixed solution are 30 mg L^{-1}.

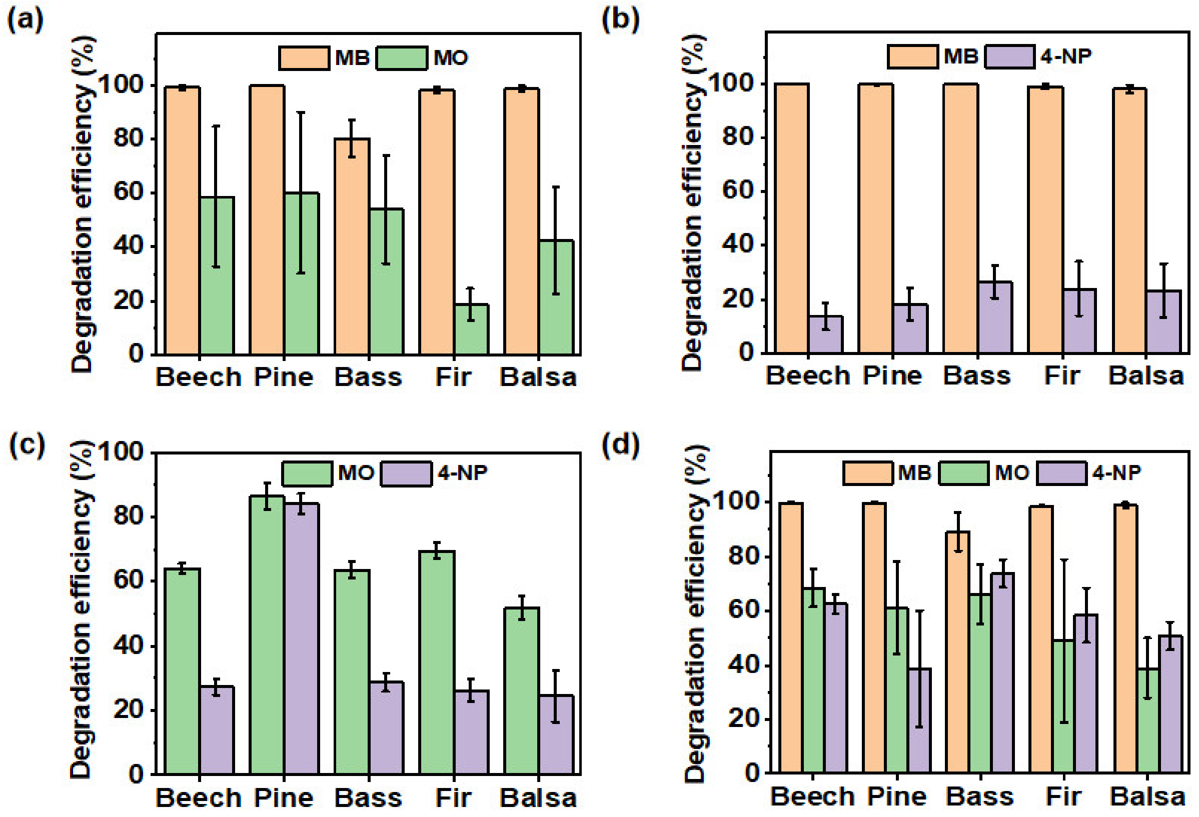

Figure S12. Degradation property of PNNW membranes to mixed organic pollutant. Degradation property of PNNW membranes to (a) MB and MO mixed solution, (b) MB and 4-NP mixed solution, (c) MO and 4-NP mixed solution, (d) MB, MO and 4-NP mixed solution. The concentrations of MB, MO and 4-NP in the mixed solution are 40 mg L^{-1}.
